# Improvements in Vulcanite

**Published:** 1882-09

**Authors:** J. B. Hodgkin


J. B. Hodgkin.

---

*Improvements in Vulcanite.*—Since vulcanite, whether right or wrong, is used so very largely, any improvements in its manipulation tending to make it more acceptable to the profession and useful and tasteful to the wearer, are in order. There were exhibited at the last meeting of the Southern Dental Association in Baltimore, a number of pieces of vulcanite, beautifully plated with gold on their palative surfaces, in a manner calculated to make such work much more acceptable to the wearer, rendering the cleansing of this part of the denture an easy task. All know how difficult is the removal of the mucous collection from the palatal surface of these plates, and it is doubtless this which is the cause of the sore mouths one often sees under such plates. In the case of these plates, as only pure gold is in contact with the mucous membrane, the poisoning theory from the mercury sulphurut is removed. So far the method seems only a success in the hands of an expert. Doubtless it could be simplified so as to render it capable of management by the average dentist. As dentists will make, and patients will wear vermillion colored plates, this invention, which we believe is patented, may put them at ease on this question. We shall watch its development with interest.

